# Efficacy and Safety of Hyaluronic Acid and Platelet-Rich Plasma Combination Therapy Versus Platelet-Rich Plasma Alone in Treating Knee Osteoarthritis: A Systematic Review

**DOI:** 10.7759/cureus.47256

**Published:** 2023-10-18

**Authors:** Md Al Amin Howlader, Ahmad Almigdad, Jannatul Ferdousy Urmi, Hassan Ibrahim

**Affiliations:** 1 Department of Trauma and Orthopaedics, Royal Berkshire NHS Foundation Trust, Reading, GBR; 2 Department of Orthopaedics, Royal Medical Services, Amman, JOR; 3 Department of Surgery, Gazi Medical College and Hospital, Khulna, BGD; 4 Department of Internal Medicine, Darent Valley Hospital, Dartford, GBR

**Keywords:** intra-articular injection, knee, osteoarthritis, platelet-rich plasma, hyaluronate, hyaluronic acid

## Abstract

Knee osteoarthritis (KOA) is a chronic degenerative disease of the joint characterized by biochemical and biomechanical alterations of articular cartilage, degradation of the joint edge, and subchondral bone hyperplasia. Nowadays, intra-articular hyaluronic acid (HA) or platelet-rich plasma (PRP) has become a popular treatment modality for treating KOA. Each treatment can be used independently or in combination. However, the efficacy and safety of combination treatment are still inconclusive, and there is a lack of high-quality level 1 studies that support using combination therapy over PRP alone.

Consequently, we conducted a systematic review to examine the effectiveness and safety of combining HA and PRP therapy versus using PRP therapy alone in KOA patients. Based on the most up-to-date evidence, the dual approach of PRP and HA therapy yields outcomes similar to PRP therapy alone in the short term, up to 12 months. Nonetheless, when considering longer-term results, particularly in the 24-month follow-up, dual therapy holds the potential to produce superior outcomes compared to PRP alone therapy. Additionally, in terms of safety, dual therapy has been associated with slightly fewer adverse events.

## Introduction and background

Osteoarthritis (OA) is a chronic and degenerative condition characterized by the deterioration of joint cartilage, resulting in stiffness, pain, and limited mobility [1]. Knee osteoarthritis (KOA) is the most commonly diagnosed form and represents a substantial portion of the global burden, particularly exacerbated by factors like obesity and an aging population [2]. The incidence of KOA has been increasing in recent decades, aligning with the trend of longer lifespans and an aging population. On a global scale, hip and knee OA are prominent contributors to disability, with KOA affecting 3.8% of the population [3]. With the aging population and rising obesity rates, the prevalence of KOA is expected to grow, leading to a significant increase in the demand for total knee replacements. Consequently, KOA imposes a significant burden on healthcare systems and societies worldwide [4-6].

At present, knee arthroplasty remains the only curative treatment for KOA, typically offered at an advanced stage of the disease [7]. The Osteoarthritis Research Society International (OARSI) recommends conservative treatments as the primary management approach for KOA, emphasizing their importance in treating the condition [8]. The American College of Rheumatology (ACR) has introduced a classification that includes both pharmacologic and non-pharmacologic treatments under conservative treatment options [9]. Non-pharmacologic treatments encompass exercise, weight loss, and diet control, which often rely heavily on patient compliance and are challenging to control [10]. Conversely, pharmacologic treatments, such as simple analgesics and non-steroidal anti-inflammatory drugs, are associated with adverse events when used over extended periods [11,12]. Therefore, it is essential to explore alternative treatment options suitable for long-term use to halt OA progression and ultimately reduce the need for surgical intervention, alleviating disability and the economic burden. Recently, both intra-articular platelet-rich plasma (PRP) and hyaluronic acid (HA) therapy have emerged as promising therapeutic advancements, with a growing body of research investigating their efficacy in treating KOA. Additionally, many systematic reviews have reported that intra-articular PRP injections, when compared to HA, can alleviate pain symptoms and improve knee function in KOA patients [13-16].

PRP is derived from an autologous blood sample and consists of concentrated platelets and growth factors [17]. These growth factors serve various functions, such as promoting local angiogenesis, modulating inflammation, inhibiting chondrocyte apoptosis, remodeling bone and vessels, synthesizing collagen, inhibiting catabolic enzymes and cytokines, recruiting local stem cells and fibroblasts to damaged sites, and inducing nearby healthy cells to produce more growth factors [18]. Therefore, this multifunctional platelet concentrate can be used to treat joint disorders, including osteoarthritis, osteonecrosis of the femoral head, cartilage injuries, and rheumatoid arthritis [19]. The initial therapeutic use of PRP was by a maxillofacial surgeon to fill cancellous mandibular defects [20]. Additionally, PRP has been shown to enhance the repair of articular cartilage injuries in patients with joint diseases by reducing harmful inflammatory factors within the musculoskeletal system [21]. It contains the necessary ingredients to stimulate repair and, to some extent, regeneration. Furthermore, one of the main advantages of platelet concentrates as a biological treatment is their cost-effectiveness, as they can be prepared through a simple centrifugation process using the patient's blood [22]. A recent meta-analysis of 21 clinical trials concluded that PRP injections provide benefits in terms of pain relief and functional improvement for KOA [23].

HA is a high molecular weight glycosaminoglycan (5-7×106 kD) that serves as a backbone for proteoglycans in the extracellular matrix and is present in various connective, epithelial, and neural tissues throughout the body [24]. It plays a crucial role in imparting viscoelasticity and lubrication to synovial fluid and the extracellular matrix [25]. HA is a vital component of synovial fluid, not only stimulating cell proliferation and migration but also providing lubrication for joint movement [26]. Osteoarthritis is associated with reduced HA within the joint cavity, primarily due to the depolymerization of endogenous HA from high molecular weight (6500-10,900 kDa) to low molecular weight (2700-4500 kDa). This process diminishes the mechanical and viscoelastic properties of synovial fluid in the affected joint, resulting in friction-induced pain [27-29]. Exogenous HA injections have been clinically used to alleviate the compromised functions of depolymerized endogenous HA in OA patients [29]. A meta-analysis of 25 clinical trials concluded that HA injection is a prominent conservative treatment option for hip osteoarthritis, offering substantial pain relief and improved function [30].

A novel treatment concept suggests that PRP and HA may promote joint repair through different mechanisms synergistically, making their combination advantageous without altering the fundamental characteristics of either product [31]. The rationale behind this approach is that since these two solutions have distinct mechanisms of action, combining them may lead to greater effectiveness than monotherapy [32]. While theoretically plausible, the increased cost of this approach necessitates clear evidence of its advantages over monotherapy before widespread recommendation. Several systematic reviews and meta-analyses have compared the efficacy of combination therapy with HA to identify a more effective treatment for KOA [33-35]. However, there is a lack of systematic reviews and meta-analyses evaluating whether combination therapy significantly improves clinical outcomes compared to PRP alone. To date, no systematic reviews have assessed the efficacy and safety profile of PRP + HA therapy compared to PRP alone for chronic KOA. Therefore, this review aims to evaluate the efficacy and safety of PRP + HA therapy compared to PRP alone in patients with chronic KOA.

## Review

The systematic review adhered to the Preferred Reporting Items for Systematic Reviews and Meta-Analyses (PRISMA) statement reporting guidelines for the systematic analysis of intervention trials [36]. It specifically focused on assessing the effectiveness of dual therapy (PRP + HA) compared to monotherapy (PRP).

Eligibility criteria

Studies were included in this systematic review if they met the following criteria: (a) intervention study; (b) non-placebo-controlled design, including randomized controlled trials (RCTs) or randomized controlled crossover trials; (c) prospective and retrospective cohort study designs; (d) participants randomly allocated to intervention and comparison groups; (e) utilized PRP and HA as interventions; (f) reported outcomes related to pain reduction and functional improvement of the knee joint, as measured by various subjective scales (as detailed in Table 1).

**Table 1:** Inclusion and exclusion criteria. TC: total cholesterol; LDL-C: low-density lipoprotein cholesterol; HDL-C: high-density lipoprotein cholesterol; TG: triglycerides; RCT: randomized controlled trials; CKD: chronic kidney disease.

Traits	Inclusion criteria	Exclusion criteria
Participants	Any age patients without multiorgan disorders	Patients with multiorgan disorders, including cerebrovascular disease, CKD, and liver failure
Intervention	Curcumin extract or nanocurcumin	Any combination therapy like curcumin + piperine and curcumin + phytosterols
Control	Placebo	Any drugs like statin
Outcome	All lipid parameters (TC, LDL-C, HDL-C, TG) and BMI	Not including all lipid parameters (TC, LDL-C, HDL-C, TG)
Study design	RCT	Cohort study, case-control study, cross-sectional study, and case series

Studies that employed other treatment routes (e.g., intraosseous) in the intervention group in combination with the intra-articular route, or those that administered local anesthetic drugs (e.g., lidocaine) alongside active treatment, were excluded. This exclusion was based on in vitro research suggesting that anesthetics might reduce platelet aggregation [37]. Additionally, studies that did not include the Visual Analog Scale (VAS) or Western Ontario and McMaster Universities Osteoarthritis Index (WOMAC) as outcome measures were also excluded from this review.

Prospective studies (RCTs) were predominantly selected for this review because RCTs are well-suited to evaluate the effectiveness of newer interventions. They help mitigate bias related to confounding factors (through the inclusion of a control group), selection bias (through randomization), and interpretation bias (through double-blinding) [38]. Trials involving any adjuvant therapy (such as micro-fragmented adipose tissue) in combination with PRP or HA were excluded. This exclusion was made to facilitate a clear differentiation between the effects of monotherapy and combination therapy.

Outcomes

The primary outcomes included the following assessments: VAS score, measured on a 10 cm scale (ranging from 0 for no pain to 10 for the worst pain) [39]. WOMAC evaluates pain, articular stiffness, and functional limitation. A higher score indicates a worse condition of the knee joint [40]. The International Knee Documentation Committee (IKDC) Subjective Knee Evaluation Form subjective score, measured on a scale of 0 to 100 (0 indicating the lowest level of joint function and 100 indicating no limitation of function) [41]. These scores were obtained at various time points: baseline, one month, six weeks, three months, six months, 12 months, and two years after treatment. The secondary outcome was the rate of adverse events.

Search strategy

We conducted a computerized search in major electronic databases, including PubMed, Cochrane Library, and CINAHL (Cumulative Index of Nursing and Allied Health Literature), for articles published up to October 2021 (as shown in Table 2). Our search followed the PICOS (Population, Interventions, Comparisons, Outcomes, and Study Designs) framework, and we used search terms related to the effects of PRP and HA on OA. Additionally, we reviewed the bibliographies of selected papers to identify additional relevant articles. We combined search outputs using Medical Subject Heading (MeSH) and keywords with Boolean operators (using "OR"). We also applied several filters to identify suitable articles, including those published in English and those published within the last 11 years. The rationale behind selecting papers from the last 11 years was to capture recent advancements in the field. In PubMed, we further restricted articles to human studies and RCTs using an additional filter.

**Table 2:** Search strategy to get relevant papers. CINAHL: Cumulative Index of Nursing and Allied Health Literature.

Database	Date	Search strategy	Limiters
Cochrane	24/10/2021	"Platelet-rich plasma" OR PRP in Title Abstract Keyword AND Hyaluronic acid OR HA in Title Abstract Keyword AND "knee osteoarthritis" OR KOA in Title Abstract Keyword - (Word variations have been searched)	Year: 2011-2021
PubMed	24/10/2021	((platelet rich plasma or PRP) AND (Osteoarthritis or degenerative joint disease)) AND (treatment or therapy)	English language, Human studies, Year: 2011- 2021, Randomized controlled trials, Clinical trial
CINAHL	24/10/2021	(Platelet-rich plasma OR PRP) AND (Hyaluronic acid OR HA) AND (knee osteoarthritis OR KOA)	English language, Last 11 years, Academic journals

Study selection

We conducted searches in PubMed, Cochrane Library, and CINAHL using keywords and applied filters. Duplicates were removed using Endnote software (Clarivate, London, UK). Subsequently, we screened the titles and abstracts of retrieved articles. Following this initial screening, studies with full-text data available were assessed, and if they met the eligibility criteria, they were included in the qualitative synthesis.

Data collection process and data items

Data were collected using a computer-based program (Microsoft Excel, Microsoft Corporation, Redmond, WA) after a thorough review of the full text of all included studies. We extracted the following information: author names, publication year, study design, country where the research was conducted, study population characteristics, mean age of the study population, the ratio of participants in the test and control groups, male-to-female ratio, dosage of the intervention and control used, and outcome measures reported in each study. Additionally, we collected data on the randomization and blinding processes, as well as information on participant attrition from the considered studies.

Risk of bias in individual studies

We assessed the quality of the included studies by evaluating their randomization and blinding methods. The risk of bias in RCTs was assessed using the Jadad score, a recognized scoring system [42,43]. For non-randomized comparative studies, we used the Methodological Index for Non-Randomized Studies (MINORS) scoring system [44]. Retrospective cohort studies were assessed for quality using the Newcastle-Ottawa Scale (NOS) [45].

Summary measures

The results of this review were summarized by calculating the difference in means between baseline and endpoint data for the outcomes of interest. The mean difference in the intervention and control groups was computed as follows: outcome = baseline data - endpoint data. The difference between the intervention and control groups was calculated as follows: difference = intervention outcome - control outcome. Since the outcome data were collected using subjective scoring systems, the results were expressed as absolute numbers.

Results

**Figure 1:**
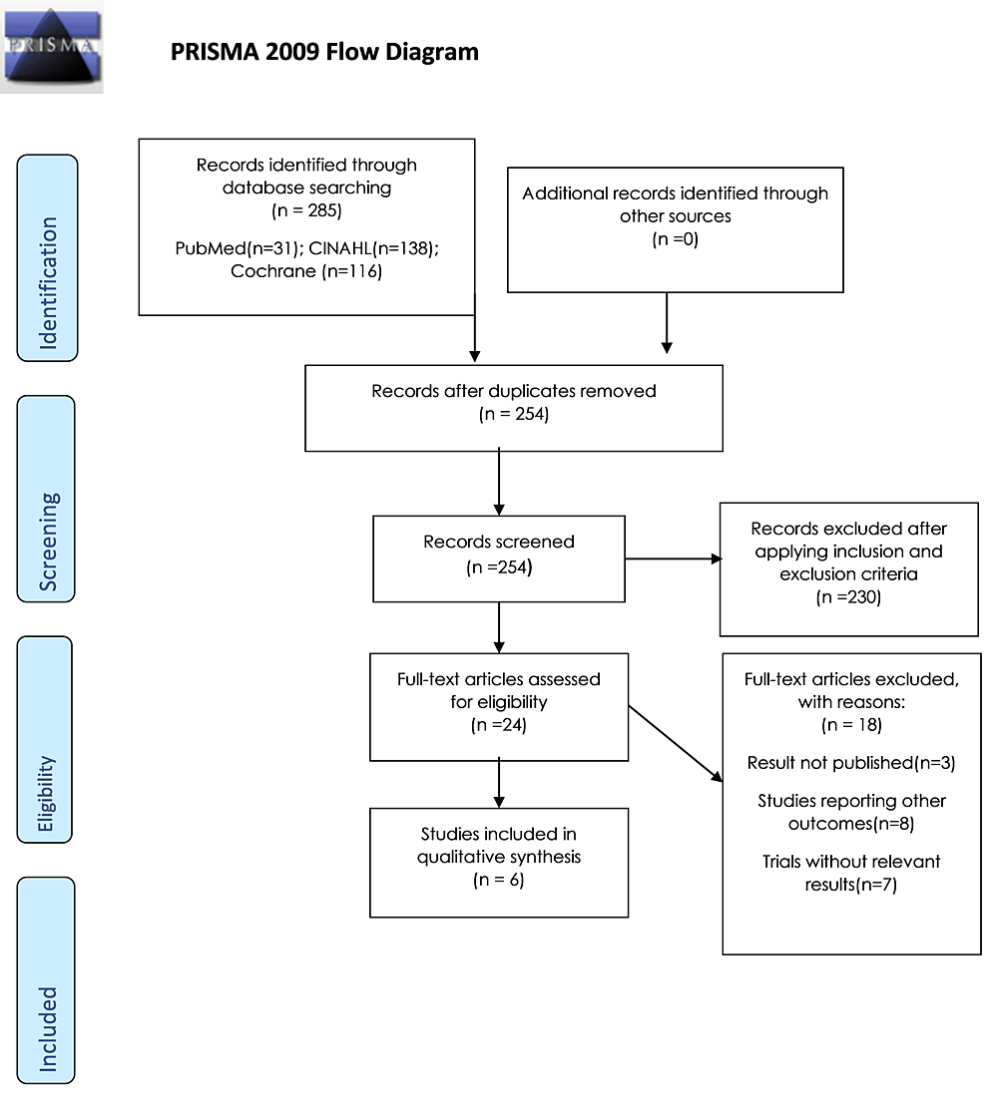
Study selection process. CINAHL: Cumulative Index of Nursing and Allied Health Literature.

Following the initial search, we retrieved 285 human trials in English from PubMed, Cochrane Library, and CINAHL on October 24, 2021. Duplicate records were removed using Endnote software, leaving 254 articles for screening. Titles and abstracts of all these articles were screened, leading to the identification of 24 articles eligible for full-text assessment. Subsequently, articles meeting the following criteria were excluded: unpublished results (3), studies reporting outcomes unrelated to the review (8), and studies lacking relevant results (7). Ultimately, six papers were selected for qualitative synthesis (see Figure 1 and Table 3).

**Table 3:** Characteristics of the included studies. PRP: platelet-rich plasma; RCT: randomized controlled trial; HA: hyaluronic acid; VAS: Visual Analog Scale; WOMAC: Western Ontario and McMaster Universities Osteoarthritis Index; TNF-α: tumor necrosis factor-alpha; IKDC: International Knee Documentation Committee; J: Jadad score; MINORS: Methodological Index for Non-Randomized Studies; NOS: Newcastle-Ottawa Scale; PDGF: platelet-derived growth factor; SLS: single limb stance; IL: interleukin; HMW: high molecular weight.

Author and date	Xu et al. (2021) [46]	Yu et al. (2018) [47]	Sun et al. (2021) [48]	Jacob et al. (2017) [49]	Lana et al. (2016) [50]	Guo et al. (2016) [51]
Country	China	China	Taiwan	International	Brazil	China
Study design	Double-blind RCT	Double-blind RCT	Single-blind RCT	Comparative study	Double-blind RCT	Retrospective study
Mean age T/C	57.9 ± 4.1/56.9± 4.2	46.5 ± 7.5/46.2 ± 8.6	60.6 ± 8.4/58.4 ± 8.1	Nil	62 ± 6.1/60.9 ± 7	61.2 ± 9.6/60.7 ± 10.1
Male:female T/C	8:20/10:20	50:46/50:54	29:18/17:22	Nil	6:27/7:29	18:45/12:51
Mean BMI T/C	21.5 ± 2.5/22.5 ± 2.3	-	25.0 ± 4.6/24.8 ± 4.1	-	29.15 ± 7.31/27.42 ± 6.89	24.2 ± 4.2/24.6 ± 3.9
Number of treatments/controls	28/30	96/104	20/20	31/20	33/36	63/63
Dose of intervention	4 ml of PRP + 2 ml HA (3 injections 2 weeks apart)	PRP: 8 ml, HA: 0.20 mg, once a week for 8 weeks	HA (3 ml) + PRP (3 ml), single dose	2 ml of PRP + 2 ml HA (HMW), single dose	5 mL of PRP + 2 ml HA (3 injections, with 2 weeks apart)	2 ml HA +2 ml PRP, 1 injection weekly for 3 weeks
PRP preparation method	Single spin	Single spin	Single spin	Double spin	Double spin	Single spin
Dose of control	4 ml of PRP (3 injections 2 weeks apart)	8ml of PRP, once a week for 8 weeks	3 ml of PRP (single dose)	2ml of PRP, (single dose)	5 mL of PRP (3 injections, with 2 weeks apart)	2 ml of PRP (1 injection weekly for 3 weeks)
Follow-up	Baseline,3, 6, 12, and 24 months	Baseline, 52 weeks	Baseline, 1, 3, and 6 months	Baseline, 6 weeks, 6 months	Baseline, 1, 3, and 6 months and 1 year	Baseline, 1, 3, 6, and 12 months
Outcomes	VAS-pain, WOMAC, adverse events	WOMAC pain, IL-17A, IL-1β, 17A, 10, 6, TNF-α. PDGF. Adverse events	VAS-pain, WOMAC, Lequesne index, and SLS Adverse events	VAS-pain, IKDC, adverse events	WOMAC, VAS, adverse events	VAS, WOMAC
Score	J-4	J-5	J-5	MINORS-18	J-5	NOS - 6 stars (fair)

Description of Studies

Among the six included studies, five are RCTs [46-50], and one is a retrospective study [51]. The total study population in these trials consisted of 544 participants, with 238 in the intervention group and 306 in the control group. Among the participants, 207 were male, and 337 were female. The age range of the treatment population was 46.5 to 62 years, while the control group had an age range of 46.2 to 60.7 years. Notably, one study did not report the participants' age range or male-to-female ratio [49]. The population characteristics of the included studies consisted of patients suffering from KOA for more than three months and diagnosed with Kellgren-Lawrence stage II-III [46,48], patients diagnosed with Kellgren-Lawrence stage 0-III [49-51], and patients diagnosed with a Karnofsky performance status of ≥80% (indicating difficulty walking independently and experiencing knee pain) [47].

The interventions varied across studies. Two studies used 2 ml PRP + 2 ml HA as the intervention drug and 2 ml PRP as the control drug [49,51]. One study treated patients with 3 ml PRP + 3 ml HA as the intervention drug and 3 ml PRP as the control drug [48]. The remaining three studies [46,47,50] applied 4 ml PRP + 2 ml HA, 5 ml PRP + 2 ml HA, and 8 ml PRP + 0.20 mg HA for the treatment group, respectively, and used PRP alone for the control group. The duration of the studies varied as well, with three studies conducted for one year, two studies over six months, and one study conducted over two years. Two studies used the double spin method to collect PRP, while the remaining five used a single spin method. All the studies provided some baseline demographic data, except for two studies [47,49] that did not mention the BMI of study participants.

All included studies were qualitatively assessed for risk of bias. The assessment of the risk of bias in each study showed that three studies met all the criteria of the Jadad score to receive the highest score of 5, while one study did not clearly report its randomization process [46]. Risk of bias assessment for Guo et al. [51] was conducted using the NOS, and one study [49] was assessed using the MINORS risk of bias assessment tool [52]. All studies reported a loss to follow-up or drop-out data, except for one study [49], which did not report this information.

Efficacy Outcome

Table 4 presents the efficacy data. Efficacy outcomes were measured using VAS, WOMAC, and IKDC scores. One study [47] did not involve VAS scoring; it only considered WOMAC scores for their outcome. In most studies, there were greater reductions in VAS scores in the intervention group compared to the PRP alone group. Only in one study [50] did the PRP control group show a more substantial reduction in VAS scores than the dual therapy group. The range of VAS score reduction in the intervention group was 1.94 to 5.9, while in the control group, it was 0.2 to 5.3.

**Table 4:** Efficacy outcome. I: intervention; C: control; D: difference; B: baseline score; F: final score; VAS: Visual Analog Scale; WOMAC: Western Ontario and McMaster Universities Osteoarthritis Index; IKDC: International Knee Documentation Committee.

Author name and date	VAS score			WOMAC score			IKDC score			
	I (B-F)	C (B-F)	D (C-I)	I (B-F)	C (B-F)	D (C-I)	I (B-F)	C (B-F)	D (C-I)	
Xu et al. (2021) [46]	2.8	0.2	2.6	15	02	13				
Sun et al. (2021) [48]	3.8	2.4	1.4	12	11.5	0.5				
Jacob et al. (2017) [49]	1.94	1.86	0.08	-	-		10.15	8.66	1.49	
Lana et al. (2016) [50]	2	2.5	-0.5	23.7	21.3	2.4				
Guo et al. (2016) [51]	5.9	5.3	0.6	25.3	25.4	-0.1				
Yu et al. (2018) [47]	-	-		23.69	15.84	7.85				

Similarly, regarding WOMAC scores, all studies reported a significant reduction in scores during the final follow-up. Four studies [46-48,50] showed a greater reduction in WOMAC scores in the combined treatment group compared to the monotherapy group. However, one study showed that WOMAC reduction was higher in the PRP single-therapy group [51]. The range of WOMAC score reduction in the intervention group was 12 to 25.3, while in the control group, it was 2 to 25.4. One study used IKDC scoring as an outcome measure, and it reported positive results for the combined treatment group [49].

Safety Outcome

Table 5 displays the safety data, including the number of patients who experienced injection site pain, joint swelling or fluid accumulation in the joint, and systemic side effects such as hypertension or proteinuria following the administration of both the intervention and control elements.

**Table 5:** Safety outcome. I: intervention; C: control; D: difference. Numbers within brackets represent the percentage within the category.

Author name and date	Injection site pain			Joint swelling			Hypertension			Proteinuria		
	I	C	D	I	C	D	I	C	D	I	C	D
Xu et al. (2021) [46]	2 (7.1)	5 (16.7)	3 (9.6)	0	4 (13.3)	4 (13.3)	-	-		-	-	
Sun et al. (2021) [48]	6 (30)	5 (20)	1 (10)	6 (30)	5 (20)	1 (10)	-	-		-	-	
Guo et al. (2016) [51]	8 (12.7)	9 (14.3)	1 (1.6)	8 (12.7)	9 (14.3)	1 (1.6)	-	-		-	-	
Yu et al. (2018) [47]	-	-		-	-		2 (2.1)	4 (3.8)	2 (1.7)	2 (2.1)	5 (4.8)	3 (2.7)
Lana et al. (2016) [50]	-	-		-	-		-	-		-	-	
Jacob et al. (2017) [49]	-	-		-	-		-	-		-	-	

Two studies [49,50] did not search for any adverse event in their study, whereas the remaining four studies mentioned the side effects of the intervention and control drug and the complications that arose after performing the intra-articular injections. Only one study reported systemic side effects, hypertension, and proteinuria, which are more prevalent in the PRP group than the dual therapy group [47]. The incidence of injection site pain was more frequent in the PRP group (19) than in the combined group (16). Regarding joint effusion, three studies reported some cases of joint swelling in both the intervention and control groups [46,48,51]. Ultrasonogram was used to find out joint pathology after treatment in most of the studies. However, more patients (15) in the PRR group were affected than the combined group (6). However, no study has reported any joint infection or serious systemic illness after treatment.

Discussion

KOA is a disease that can cause disability by affecting the locomotive function of the lower extremities, hampers the quality of life of patients, and has a detrimental effect on the physical and mental health of middle-aged and elderly people [53]. The pathogenesis of KOA is still unclear. Several hypotheses have been proposed, but still, there is not a clear etiological factor or disease pathway of its natural course that has come to light, which explains not having an effective conservative treatment [54]. Hence, depending on those hypotheses, multiple treatment strategies have been developed and tested; some of them become more effective than others, but eventually, all are aimed at decreasing pain, increasing function, and halting the requirement for a surgical joint replacement [55]. However, to date, surgical treatment is the only treatment for severe KOA [56]. Intra-articular injection of PRP or HA become compelling in treating KOA, which has evoked strong interest from many clinicians, and in-depth research has been conducted [57,58]. The basic science rationale of combining PRP + HA is promising. These two solutions have unique and complementary effects on the OA environment. Combination therapy aims to titrate these unique effects and maximize the therapeutic potential. Perhaps this combination may reduce the incidence of unavoidable surgery, complications risks, and the financial burden of surgery. However, to date, no study has analyzed the cost-effectiveness of combination therapy for treating KOA. In addition, no study has compared the efficacy and safety of this combination with PRP therapy alone. That is why we aimed to conduct this systematic review to get an overview of the potential pharmacologic intervention that may be used as an effective treatment strategy to halt disease progression and will help to reduce the financial burden of surgery.

In this review, no risk of bias was found in three studies after calculating the Jadad score [47,48,50]; one study [46] did not mention the randomization or blinding process. One study [51] was marked as fair enough (six stars) by NOS and another study [49] was marked as a good quality paper (scored 18) by MINORS scoring (good quality range: 16-24) (Table 3). Therefore, the studies included in this review exert less risk of bias, which signifies the reliability of the review results.

In this review, the HA + PRP combination therapy improved the outcome of reducing joint pain and stiffness, increasing joint mobility, and functional improvement of lower limbs than PRP therapy alone. Here, the included studies reported significant differences between baseline and endpoint follow-up in VAS scores, WOMAC scores, and IKDC scores in both the intervention and control groups (Table 4). PRP shows noticeable improvement in the subjective scoring systems. However, the effect shown by the combination group is still higher than that of the PRP alone group. In short-term follow-up, the PRP and combined treatment groups showed almost similar efficacy in reducing pain in most studies. However, in one study [46], a significant difference in pain-reducing capacity was noted. Xu et al. [46] found similar results with other studies included in this review when the follow-up was taken at six-month and 12-month periods. However, at 24-month follow-up, the pain-reducing capacity of PRP significantly deteriorated, and the pain scale appears to be the same as the baseline score. A five-year double-blinded RCT was conducted to compare the long-term efficacy of PRP and HA treatment in terms of pain-reducing capability, which they found at 24 months and the final follow-up at 64.3 months. Both PRP and HA yielded no significant results; rather, the scores are similar to the baseline score [59]. On the contrary, a recent systematic analysis compared 15 RCT results and concluded that PRP injection therapy is a safe treatment with the potential to provide symptomatic benefits for OA in the short-term period (six to 12 months) and is more efficacious than those of HA [60]. This could be due to dilution in the amount of PRP growth factors or HA degradation over time, which is responsible for not having the desired therapeutic effect for a long duration. If additional treatment modalities were adopted, such as the continuation of the intra-articular injection on a six-month or 12-month basis, then the outcome would have shown promising results.

Apart from the VAS pain score, this study also evaluated two important patient-reported outcome measures: WOMAC score and IKDC score. This study found better WOMAC and IKDC scores in patients who received PRP-HA dual therapy compared to patients who received PRP-alone therapy (Table 4). This implies there is improvement in all three domains of WOMAC: reduction in joint pain, increase in the range of joint movement, and increase in the functional capacity of the lower limb [40]. Besides, IKDC score improvement implies a reduction of knee symptoms (pain, swelling, and stiffness), an increase in daily activities and sports, and an improvement of current knee function compared to prior knee function [61]. Five studies reported that the WOMAC function score is significantly better for the dual therapy group at baseline and final follow-up compared to the PRP-alone group (Table 4). This finding is corroborated by the systematic study of Zhao et al., who noted that PRP and HA dual therapy achieved better WOMAC scores at 12 months compared to PRP-alone therapy [62]. In addition, one study [49] evaluated the IKDC score for knee function assessment, which showed better improvement in physical function for the dual therapy group at the final follow-up compared to the PRP-alone group. However, the notable finding is that these two scores are also quite similar in intervention and control groups in those studies conducted for six to 12 months, which was also reflected in the VAS scoring system (Table 4).

In the context of cost-effectiveness, few studies compared the cost-effectiveness of different non-surgical [63,64] and surgical treatment [65,66] strategies for KOA treatment; however, no direct cost analysis study has been done between intra-articular injections and surgical treatments. A study conducted in France in 2016 [67], concluded that intra-articular PRP-based therapy is cost-effective with regard to the intra-articular HA in one-year follow-up. They reported that the average cost per quality-adjusted life year (QALY) is, respectively, around €761.5 for HA and €761.7 for PRP, and the incremental effectiveness of PRP is 0.166 QALY with an incremental cost of €126.21 [67]. A study [68] conducted in the USA concluded that incremental cost per total knee replacement (TKR) was $20,133 in 2007, which is 14 to 15-fold greater than the cost of conservative treatment. In addition, a study in Canada [69] evaluated the surgical cost (arthroplasty) for five patients, and that was $10,476.53.

On the contrary, they found that the average cost of medical management over the two-year time frame (including the five who ultimately proceeded to surgery) was $925.43, more than 10 times less than the surgical treatment. Although combining PRP with HA requires additional costs, it is outweighed by additional benefits in terms of analgesic potency [67]. Hence, the major advantage of PRP and HA, in addition to quality-of-life improvement, could delay the requirement of TKR and, therefore, an economically attractive treatment option compared with operative treatments.

In the context of safety, both the combination and monotherapy showed some incidence of adverse effects, which were temporary. There is no significant difference in the incidence of local adverse events rates in the PRP alone group with the dual therapy group (Table 5). Three of the studies reported injection site pain and joint swelling after the procedure in very few participants, lasting two to five days (Table 4). This may be due to some pro-inflammatory cytokines in the PRP and the inflammation in response to minor tissue injury that occurred during the insertion of treatment agents into the joint space [70]. Another possible cause for the combined treatment group may be the body's immune response to HA [71]. However, no study has searched for the reason behind these adverse reactions. The etiology could have been ruled out by synovial fluid analysis for eosinophil count (immunologic sensitization) and culture (infectious agents) [72].

One study reported treatment-emergent adverse events in the form of hypertension and proteinuria and found that ≥10% of the study population was affected at the end of the study [47]. Theoretically, the presence of leukocytes in PRP increases pro-inflammatory activity by expressing catabolic cascades and releasing inflammatory markers, which can lead to this condition [73-75]. However, this is still unclear and needs further research to determine the actual cause.

Characteristics might still be responsible for different results, together with many aspects such as the activation of PRP and the use of different application protocols, injecting in different compartments of the knee joint, different molecular weights of HA, cross-linking, sources, volume, and how they are mixed with the PRP and also the mixing process of PRP and HA [76,77]. Evidence suggests that PRP's effectiveness depends on single spin vs. double spin and leucocyte-poor PRP vs. leucocyte-rich PRP [59,78]. Two studies included in this review used the double-spin method to prepare PRP, and the remaining four used the single-spin method (Table 3). However, no study used photo-activated PRP preparation for intra-articular use, which is also an important factor of PRP pharmacokinetic effect [79]. BMI is an important risk factor for developing osteoarthritis in weight-bearing joints [80]. BMI has been identified as an independent risk factor for treatment failure in KOA treated with PRP, and BMI > 25 has the highest influence on the treatment outcome [81]. There was a significant discrepancy in the BMI range of participants included in this review (Table 1). Another important factor is that most of the female participants included in this review have the age range of 46 to 60 years, which is a perimenopausal state, and this is associated with the onset and progression of OA in women [82]. No study has reported the hormonal drug intake status of the female participants in their patient characteristics or considered it a patient selection criterion for their study. A recent systematic analysis shows that women who are undergoing hormone replacement therapy (HRT) for menopause have a significant improvement for thumb base osteoarthritis (TBOA) treated with PRP or HA therapy than those who are not [83]. These are a few crucial factors for future clinical and research efforts that should be considered.

There are certain unavoidable limitations in this review. First, two articles were non-RCTs, which may have led to the heterogeneity of the combined indicators. Second, the follow-up was short, with the longest follow-up period being two years, and the long-term efficacy and safety of PRP combined with HA could not be evaluated. Third, one study did not directly compare the efficacy and safety of the intra-articular injection of PRP combined with HA with those of the intra-articular injection of PRP alone. Fourth, the outcome measures are based on only subjective data. No study has used MRI to investigate the intra-articular environment, such as articular cartilage, meniscus lesion, and synovial membrane thickness, which may provide necessary objective data regarding the outcome of each therapy.

## Conclusions

This systematic review suggests that the combined PRP-HA therapy and PRP-alone therapy may yield promising clinical effects on KOA in the short term (six to 12 months), with better outcomes observed in the combined therapy group over a long-term period (two years). Additionally, concerning the incidence of adverse effects, intra-articular injection of PRP combined with HA shows similarities to the adverse effects associated with intra-articular injection of PRP alone. Therefore, the safety profiles of both treatment regimens are comparable. However, it is worth noting that the systemic side effects of PRP-alone therapy are higher than those of combined therapy, implying a preference for the combined therapy approach. Consequently, monitoring the clinical effects and tolerability of patients undergoing PRP or HA treatments in clinical trials or clinical practice is of paramount importance.

Nevertheless, long-term follow-up RCTs with larger sample sizes are necessary to determine the sustained efficacy and safety of combined treatments. As such, it could be considered a suitable conservative treatment for KOA, potentially reducing the financial burden associated with arthroplasty surgery.
